# The use of technology for arts-based activities in older adults living with mild cognitive impairment or dementia: A scoping review

**DOI:** 10.1177/14713012221127359

**Published:** 2022-10-04

**Authors:** Jennifer MacRitchie, Georgia A. Floridou, Justin Christensen, Renee Timmers, Luc de Witte

**Affiliations:** Department of Music, 7315 The University of Sheffield, UK; Healthy Lifespan Institute, 7315 The University of Sheffield, UK; Department of Music, 7315 The University of Sheffield, UK; Department of Music, 7315 The University of Sheffield, UK; Healthy Lifespan Institute, 7315 The University of Sheffield, UK; Centre for Assistive Technology and Connected Healthcare (CATCH), 7315 The University of Sheffield, UK

**Keywords:** arts, technology, dementia, mild cognitive impairment, scoping review

## Abstract

For older adults living with mild cognitive impairment or dementia, creative arts-based activities can offer many benefits from enjoyment as leisure/recreation to an avenue to maintain cognitive, social and emotional wellbeing. With growing interest and recognition that technology could have potential to assist in delivering these activities in more accessible and personalised ways, a scoping review was undertaken to systematically examine the scientific literature for technology-assisted creative arts activities for older adults living with dementia. We searched PubMed, PsychINFO, Web of Science, Scopus and ACM Digital Library databases using keywords centering on population with dementia, an intervention using technology, and a context of creative arts, with no restrictions on the type of outcome measured. We retrieved 3739 records, with an additional 22 from hand-searching. 51 full-text articles met the inclusion and exclusion criteria. Findings of the review indicate technologies principally being designed for music activities (listening, and music-making), as well as storytelling and visual arts. The majority of devices were custom-made, with studies mainly reporting on validating the success of the device/intervention. This suggests most work in the field is currently at prototyping stage, although a few devices are now commercially available. Recommendations for future research includes involvement of participants reporting on their previous experiences in the arts and how this influences co-design choices, and inclusion of different severities of dementia in the participant/co-design group. Furthering device development past prototyping stage as well as collaboration between teams would enable comparisons to be made across different types of devices used for the same activity, and comparisons across arts-based activities that could lead to cross-disciplinary outcomes for the design of creative arts-based assistive technologies.

## Introduction

Engagement with arts-based activities is ubiquitous across the lifespan. Positive effects are found across leisure and creative expression, to impacting cognitive, social and emotional wellbeing for the general population (Dingle et al., 2021; Fancourt et al., 2021; Gordon-Nesbitt & Howarth, 2020) as well as specifically for older adults (Fancourt et al., 2020; Rogers & Fancourt, 2020; Tymoszuk et al., 2020). Whether engaging in the arts as a novice, or in continuation of an identity developed over the lifespan as an artist, musician, or simply someone who appreciates attending concerts or visiting museums, facilitating availability and participation in these activities (and consequently “belonging” to these social groups) throughout changes in a person’s life is thought to contribute to self-esteem, feelings of control, and meaning via the social identity approach (c.f. Dingle et al., 2021).

Over 50 million people are affected by dementia worldwide, with age being one of the predominant risk factors (https://www.dementiastatistics.org/statistics/global-prevalence/). In the UK alone 850,000 live with dementia and predictions are that this number will increase sharply in the decades to come (Wittenberg et al., 2019). People who live with cognitive impairments experience symptoms that have considerable impact on their everyday life which results in a diverse range of needs. Medical and clinical care has been the main priority for supporting and improving their everyday functioning. However, it is acknowledged that engagement in meaningful activities can support psychosocial needs, outcomes that have been indicated as a high priority by people living with dementia (Reilly et al., 2020).

Due to their nature as non-pharmacological interventions, arts-based activities that cater for older adults living with cognitive impairment (from mild cognitive impairment through to early, mid and later stages of dementia) are becoming more widespread (Cutler, 2020; The Commission on Dementia and Music, 2018) with reported benefits for decreasing depression and agitation (Van der Steen et al., 2017; Zhang et al., 2017), improving mood and engagement (Lourida et al., 2020) and possible cognitive effects in memory, concentration and communication (Young et al., 2016). Availability of these types of activities may depend on several contextual factors such as local provision of expertise (e.g., community artists, art/music therapists, or volunteers), and resources (internet connection, digital devices, transport, dementia-friendly venues), differing also in terms of being situated either in the community or in residential care. Although activities such as those involving music are fairly pervasive even in residential care, residents often note a decrease in access to music upon moving into a residential care home (Paolantonio et al., 2021). It is noted that tools and technologies could assist in making these activities more accessible, efficient and effective (Garrido et al., 2020), an aspect that has been intensified by the recent COVID-19 lockdown (Cutler, 2020; Dowson et al., 2021). The current scoping review specifically addresses the use of technology for arts-based activities for people with mild cognitive impairment and dementia as well as their carers.

### What constitutes arts-based activities?

Several definitions exist about what constitutes an arts-based activity. Arts engagement typically refers to various forms of activities that are creative, either in an active, (e.g. painting, dancing, music-making) or receptive manner (such as attending a concert, visiting an art gallery, watching a theatrical play). Engaging with the arts, whether active or receptive, can be a multisensory experience often involving various auditory and/or visual elements. Although definitions may also encompass wider creative activities more in line with leisure or recreational activities (e.g. cooking, gardening, involvement in social clubs; c.f. Fancourt et al., 2021), for the purposes of this scoping review, we focus on those activities which foreground an artistic medium whether auditory (sounds, music or speech; c.f. poetry, storytelling), visual (painting, sculpture, crafts), or audio-visual (drama, dance). Arts engagement is also something that can be enjoyed on an individual basis, or as part of a group. These contextual elements (for music this is detailed in Brancatisano et al., 2020) as well as the presence (and role) of a facilitator (Krause et al., 2019) may shape an individual’s experience of the arts-based activity.

### Design, assistive and everyday technologies for the arts

According to a recent review, design interventions involving playful artefacts, physical games, multisensory experiences, new technologies and services for people living with dementia has progressed in four main areas: (i) reminiscence and personhood, (ii) social integration and ‘living in the moment’, (iii) independent and assisted living, and (iv) cognitive and physical stimulation (Tsekleves, 2021). When it comes to the use of technology within these interventions, despite gaining popularity in the past two decades, particularly for reminiscence therapy (Lazar et al., 2014), adoption by policy and practice has been slow (Astell et al., 2019). Assistive technology is an umbrella term covering the systems and services related to the delivery of assistive products and services that maintain or improve an individual’s functioning and independence, thereby promoting their well being (World Health Organization, 2018). Assistive technology used by people living with dementia and their carers can be categorised into technologies for assisting daily living, safety, telecare, engagement, social participation and leisure. Although there is a growing recognition and interest in using assistive technology for leisure and cultural or arts-based activities, as of 2018, very few devices have been dedicated to these pursuits as of yet (Klimova et al., 2018). The increasing “technification” of older adulthood, i.e. the increasing policies, funding and research aimed at innovating technology solutions to the needs of older adults (Peine, 2019) and growing engagement with everyday computer, smartphone and tablet devices for accessing photos and music online (Sweeney et al., 2021) indicates higher technology use in the future. This is notwithstanding the impact of the recent COVID-19 pandemic in accelerating the use of digital strategies through telemedicine (Cuffaro et al., 2020) or arts practices for people living with dementia (Cutler, 2020; Dowson et al., 2021). In addressing the gaps in arts-based technology design, conducting design research that centres the lived experience of people living with dementia (i.e. co-design practices) can enable the dementia voice to be a part of defining the scope of a problem or need (i.e. what arts activities do people with dementia want to participate in?), as well as contributing to any solution offered (Nygård et al., 2019; Tsekleves & Keady, 2021). Technology design work in this space reviewed by Tsekleves (2021) reports a general aim of designing tools that develop personalised interactions for people living with dementia, in accordance with an individual’s needs and preferences.

### Scope of the current review

With this review we turn focus onto particular instances where technology has been adapted or designed to enhance creative arts participation for older adults living with dementia. We believe a scoping review is necessary to draw together the various developments in technology that have been reported for different art forms and to devise future directions for the design and implementation of these tools. Accordingly, the main aim of the presented scoping review was to document the research studies using technology for creative arts with people living with dementia. Within this aim, we investigated (a) the characteristics of the arts-based activities, (b) the types of technology used and (c) how these intersected in a particular context. Contextual elements considered here also included the social context of the activity, presence of facilitators, as well as the direct involvement of people living with dementia in design and/or testing as these features may have had an influence on various design choices and their potential use for particular groups. The extent to which a piece of technology could offer choice, or be adapted towards the user’s individual needs was noted.

## Method

Scoping reviews are suitable for investigating broad topics with the intention of comprehensively and systematically mapping the relevant literature and identifying key themes as well as gaps in this literature. Unlike systematic reviews, scoping reviews do not have pre-specified study designs or strict exclusion/inclusion criteria. Most commonly, scoping reviews are narrative and descriptive for the purposes of providing an overview rather than synthesising the individual studies or judging their quality (Arksey & O’Malley, 2005; Levac et al., 2010). This scoping review was conducted using the methodological framework of Arksey and O’Malley (2005). The framework is divided in five stages and the review process is presented and described accordingly.

### Stage 1: Identifying the research question

The research questions that guided the review were the following:

1. What are the arts-based activities being delivered through technology for older adults living with dementia or mild cognitive impairment?
2. What types of technologies are being used to enhance arts-based activities for older adults living with dementia or mild cognitive impairment?
3. To what extent do these technologies offer choice or adaptability to the individual user?
4. How do these technologies and arts activities intersect and in what context are they delivered including country, participants and social context?
5. How are the outcomes of using these technology-assisted arts-based activities measured?

### Stage 2: Identifying relevant studies

We conducted a systematic search of the literature in PsychINFO, PubMed, Web of Science, Scopus, and ACM on 19 March 2021, using a search strategy and search terms that were identified a priori. We included keywords that were used in related papers (e.g. reviews, studies) about arts and/or technology (Creech, 2019; Fancourt et al., 2021; Sweeney et al., 2021; Wang et al., 2020), keywords identified in key publications and discussions with researchers, and the Arts and Humanities Research Council report “Understanding the value of arts and culture: The AHRC Cultural Value Project” (see Table 1). The objective was to be highly inclusive in definition of arts, technology and dementia, before trimming down the selection of articles as a next step. There were minor variations as appropriate for each database.

**Table 1.** Search strategy for databases.

Search block	Search terms
Population	TITLE OR ABSTRACT: (Dementia OR alzheimer* OR “mild cognitive impairment” OR MCI) AND
Arts-based activities	TITLE OR ABSTRACT: (Creativ* OR leisure OR “Cultur* activit*” OR “leisure Time” OR Recreation* OR “Self-expression” OR improvis* OR Art OR Arts OR “Arts-based” OR “Art-based” OR “Art-viewing” OR “Arts-viewing” OR “participatory arts” OR “participatory art” OR “performing arts” OR “performing art” OR “Community arts” OR “Community art” OR “Sound installation” OR music* OR “musical instrument*” OR singing OR choir* OR dance OR dancing OR drama OR “dramatic art” OR “dramatic arts” OR theatre* OR theater* OR Concert* OR gig OR gigs OR festival* OR Acting OR museum* OR exhibition* OR galler* OR Craft* OR “visual art” OR “visual arts” OR photograph* OR film* OR film-making OR “film making” OR animation* OR “motion picture” OR “motion pictures” OR video-making OR “video making” OR drawing OR painting OR pottery OR jeweller* OR Sculptur* OR Storytelling OR poem* OR “literary art” OR “literary arts” OR poetry OR “Creative writing” NOT “State of the art” NOT State-of-the-art NOT State-of-art NOT “State of art” NOT “drawing on” NOT “Clock drawing” NOT Clock-drawing NOT entertainment NOT “Acting on” NOT “Creative common*”1) AND
Technology	TITLE OR ABSTRACT: (Technolog* OR “Assistive Technolog*” OR “supportive Technolog*” OR “health Technolog*” OR gerotechnolog* OR gerontechnolog* OR technophilia OR “interactive technolog* application*” OR App OR digital OR Touchscreen OR “touch screen” OR “Tablet computer*” OR “Tablet device*” OR “digital Technolog*” OR “digital Computer*” OR Computer* OR iPad* OR “mobile device*” OR “mobile Application*” OR Smartphone* OR “smart phone*” OR “mobile phone*” OR “Cell phone*” OR “virtual Reality” OR VR OR “immersive virtual Reality” OR “augmented Reality” OR “Sensor based technolog*” OR “Sensor-based technolog*” OR “multisensory environment*” OR “Computer simulation” OR “Computer application*” OR “artificial intelligence” OR AI OR “new media” OR internet OR “web site*” OR Website* OR web OR device* OR Software OR “Computer game*” OR “digital game*” OR “video game*” OR Robot* OR “human Computer interface*” OR “human Technolog* interaction” OR “human Robot interaction” OR “human Computer interaction” OR “human machine System*” OR “human-centered computing” OR interface* OR “user interface*” OR “user-computer interface*” OR electronic* OR “Self-help device*”)

Note: The terms were adjusted depending on the database (e.g., MeSH terms and truncation were used where appropriate).

Reasoning behind selection of NOT terms: “state of the art” too broad (synonyms: state of art/state-of-the-art/state-of-art), “clock drawing” (clock-drawing) refers to cognitive tests and not drawing as an activity, “drawing on”most frequently refers to the phrasal verb rather the activity, “entertainment” is too broad, “acting on” most frequently refers to the phrasal verb rather the activity; creative commons. Search terms dropped: not sound but sound installation as sound is too general, for example sound classification/analysis for dementia detection, sensor* as it was too general (e.g., biosensor, sensorimotor, sensory), tablet as it frequently refers to pills but kept tablet computer and tablet device; cultur* as very frequently it refers to blood/cells/mouse cultures and replaces with cultur* activit*.

We restricted the search in the scientific databases to titles and abstracts and used controlled vocabulary for PsychINFO (Thesaurus) and PubMed (MeSH). The search strategy was reviewed and approved by a librarian of The University of Sheffield. To identify any additional potentially relevant papers we hand searched the conference proceedings since 2010 of the European Society for the Cognition of Music (ESCOM), New Instruments for Musical Expression (NIME), International Conference on Music Perception and Cognition (ICMPC), as well as IEEE Xplore, and the Google Scholar accounts of prominent authors related to the topic of the review, and reference lists.

The records that were eligible for inclusion in the review were full-text, peer-reviewed journal articles, conference proceedings papers, and conference proceedings published in book series. Publications such as reviews, editorials, not original research, and the abstracts of conference proceedings were excluded. The search was restricted to recent publications in English, where recent was defined as between January 2010 and 19 March 2021, when the article search was completed. Initial work in developing technology for arts-based activities was completed by Alm and colleagues before this period (Alm et al., 2007) through the Living in the Moment project. This initial system allowed users to explore and interact with different virtual environments including visiting a museum. Development of a pilot digital musical instrument specifically for those living with dementia was also reported in (Riley et al., 2009; see also for a brief review of leisure-assisting technologies to this date). The current review took 2010 as the lower limit as this year marked the launch of the first generation iPad tablet and a general increase in interest for this type of technology use for older adults (see Hung et al., 2021).^1^ We did not include or exclude studies on the basis of their research design or methodologies. All studies were expected to address the development of technology for arts engagement with older adults living with dementia or mild cognitive impairment and to have engaged directly with this population at some stage of the research. Studies including younger adults with early onset dementia or mild cognitive impairment were excluded.^2^

As familiarity with the available literature increased, we refined our inclusion and exclusion criteria post hoc. Inclusion criteria meant that studies were included that (a) described any type of technology, with the exclusion of common everyday technologies older than the 10-year search period that had not been further adapted (e.g. CD player, or TV), (b) either the main focus was arts-based or when this wasn’t the case, the art form was in the foreground of the activity. Studies were excluded if they (a) described participants, arts-based activities, and technologies in very minimal detail (e.g. a paper which details the participants as “care home residents” but did not specify incidence or stage of dementia; where the activity was described as arts-related but no further details are provided; where technology was reported minimally but no details were provided), (b) solely presented perceptions of carers on how people living with dementia or mild cognitive impairment were likely to experience the usage of technology, (c) described the art form in the background of the activity. For example, papers that were excluded reported activities that used music and/or photos in the background of serious games or in reminiscence therapy to stimulate memory and conversation (c.f. Lazar et al., 2014 for review), (d) reported mixed results or observations of people living with dementia or mild cognitive impairment along with healthy older adults, (e) described a context where participants were not directly interacting with technology, or (f) described technologies that were not tested and were presented as theoretical ideas about the development of technologies (i.e. prospective use articles).

### Stage 3: Study selection

**Figure 1.**
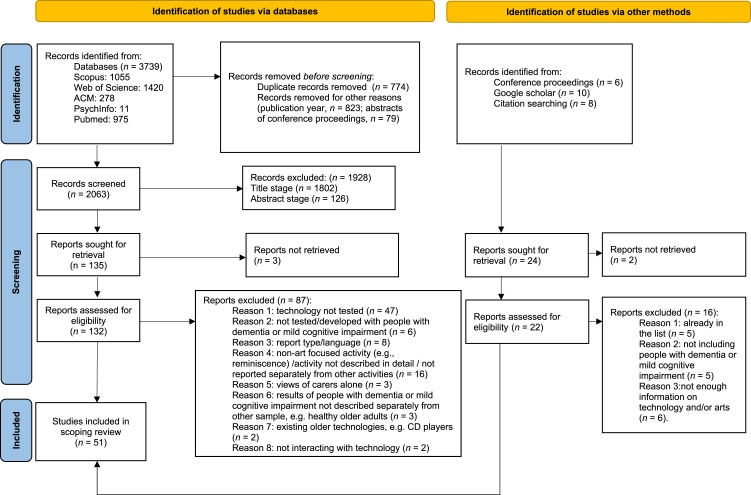
PRISMA 2020 flow diagram of literature search.

First, we imported the results from the databases to Mendeley and JabRef and we removed the duplicate records. Then the titles of the records were added in a google sheet. Next, co-authors JM and GF independently screened the titles of the records and rated them on a 3-point scale (0 = “not relevant”, 1 = “maybe relevant”, and 2 = “certainly relevant”). The sum (and absolute difference) of these scores indicated the precision of selection criteria. Records with a score of ≥2 entered the next stage for abstract screening. In cases where there was a clear disagreement between the authors’ scores (when the absolute value of the difference of their scores was 2) co-author <blinded for review> acted as a third assessor. The same process was repeated at the abstract screening stage. Records that passed the abstract stage were entered for the full text analysis stage (see Figure 1).

### Stage 4: Charting the data

The main information extracted from each publication detailed three main aspects: (i) demographics such as the authors, country, characteristics of participants involved (cognitive impairment or dementia as well as severity), the sample size and related sample demographic information (age and gender), (ii) art-form characteristics detailing the type of the arts-based activity (music, storytelling, art etc.), the form of engagement of the participants (active, receptive or mixed), the social context that was involved when the activity was taking place (solo, group), and the presence of a facilitator (yes/no), and (iii) technology characteristics including type (tablet, robot, custom device etc.), mode (whether delivery was audio, visual or audiovisual), the extent of user involvement (testing, co-design or both), as well as a description of how this served to deliver or enhance the arts-based activity.

### Stage 5: Collating, summarizing, and reporting the results

To summarize findings (as seen in Table 2), the publications were grouped in order of frequency according to (1) the type of the arts-based activities they described, then (2) the types of technology that were utilised. In the former, film-making and storytelling activities appear next to each other in Table 2 due to similarities in the type of activity. In the latter, we categorised technologies in terms of whether they were custom-made devices, portable media players, applications for tablet/smartphone/computer, virtual reality, robots, or web-based applications, and then presented according to frequency.

**Table 2.** Types of technology use in art-forms for people living with mild cognitive impairment or dementia, identified with supporting references.

Author(s), year, country	Sample characteristics (mild cognitive impairment, dementia type and/or severity), sample size, age (A), gender (G) where reported	Art-form	Engagement type	Social context	Facilitator	Technology type	Delivery mode (au - audio; AV - AudioVisual; V - visual	User involvement	Adaptability	Output measures (Beh. - Behavioural; Cog. - Cognitive; Conn - connection; comm - communication; explor. - Exploration)	Description of technology use in art-form
Seymour et al. (2017); Canada	Person with dementia, *n* = 8;	Music listening	Receptive	Solo	Yes	Custom device	Au	Co-design	Interface can be switched to match user’s needs	Feasibility	Tangible music player (AMI: Adaptable music interface) that fits around iPad and can be reconfigured to the user’s specific needs by either adding or removing physical buttons, switches, or rotary encoder.
Thoolen et al. (2019); Netherlands	Person with dementia	Music listening	Receptive	Solo	Yes	Custom device	Au	Co-design	Interface can be switched to match user’s needs	Feasibility	Sentic is a tailored music-playing device/interface (physical and digital). It has a record player base and three modules/plug-ins (discrete interface, explorative interface, no interface) adaptable to the skills of the person, allowing person with dementia to interact with their personal playlist(s).
Hsu et al. (2019); tawain	Person with dementia, *n* = 10; caregiver interviewees *n* = 2	Music listening	Receptive	Solo	Yes	Custom device	Au	Testing	n/a	Feasibility, BPSD (Beh.)	KKBox app used as playlist management for music to be played on smartphone or other electronic device (tablet, computer). Physiological measurements taken from fitbit to determine appropriateness of music for person with dementia.
Houben, Brankaert, et al. (2020); Netherlands	Person with dementia, *n* = 19; professional caregivers, *n* = 16	Music listening	Receptive	Solo w/group	Yes	Custom device	Au	Testing	Possibility to upload personally relevant stimulus materials	Feasibility, interaction (Conn.)	Vita is an interactive ‘pillow-like’ sound player that gives person with dementia access to environmental sounds through touch interaction.
Bennett et al. (2016); UK	*n* = 8; A: Range “mid 70s - early 90s”, G: 7F	Music listening and audio listening	Receptive	Solo	No	Custom device	Au	Testing	n/a	Feasibility	Rocking chair embedded with speakers, driven by iPod touch device playing sound and mobile device accelerometers that sense motion to create sounds when moved.
Cunningham et al. (2019); UK	Person with dementia (vascular or AD), *n* = 14 (cohort 1: *n* = 6, cohort 2: *n* = 8); A: Range 69–97 (*M* = 84.60, *SD* = 8.69), G: 8F; care home staff;	Music listening	Receptive	Solo and group	Yes	Application for tablet	Au	Co-design and testing	Personally relevant stimulus materials used	BPSD (Beh.), QoL	Musical Tracks is a musical mobile app available on android that associates musical tracks with daily tasks through song-task association in order to support daily routines.
Nayer et al. (2014); Australia	Person with dementia (mild), *n* = 7;	Music listening	Receptive	Solo	Yes	Application for tablet	Au	Co-design	Possibility to upload personally relevant stimulus materials	Feasibility	Touchscreen multimedia ‘memory box’ device with large touchscreen buttons and redundant physical buttons. Allows access to personalised music, videos, photographs, and pre-recorded messages and is intended for independent use.
Peeters et al. (2016); Netherlands	Person with dementia dyads with relatives (spouse, child), *n* = 5; A: Range 50–80 s, G: 5M	Music listening	Receptive	Solo (w/carer or group option)	Yes	Application for tablet and smartphone	Au	Co-design	Possibility to upload personally relevant stimulus materials	Feasibility, QoL, interaction (Comm.)	Music ePartner (touchscreen application) that allows access to annotated playlists, music and picture albums, and picture slideshow.
Gilson et al. (2019); USA	Person with dementia (non-normative memory loss is not recorded, but residential facilities self-selected participants who fit this criteria. It is assumed they all have dementia), n=1089 separate sessions, unclear on number of participants	Music listening and other activities	Receptive	Solo	Yes	Application for tablet	Au	Testing	Possibility to upload personally relevant stimulus materials	QoL	Person with dementia use tablet (e.g. iPad) that has various apps (e.g. YouTube, google, personal playlist, personal photos or videos, puzzles) to improve mood.
Lancioni et al. (2015); Italy/USA/NZ	Person with Alzheimer’s (mild or moderate; MMSE range 19–22), *n* = 3;	Music listening	Receptive	Solo	No	Computer/laptop (w/software, speakers, camera)	Au	Testing	Possibility to choose from pre-existing list	BPSD (Beh.)	A laptop computer with an amplifier, a microswitch (handheld pressure device), and software that enables person with Alzheimer’s to choose preferred music from different music options.
Lancioni et al. (2014); Italy/USA/NZ	Person with Alzheimer’s (moderate; MMSE range 16–20), *n* = 4; A: 75–89 (*M* = 81), G: 3F	Music listening	Receptive	Solo	No	Computer/laptop (w/software, speakers, camera)	Au	Testing	Possibility to choose from pre-existing list	BPSD (Beh.)	A laptop computer with an amplifier, a microswitch (handheld pressure device), and software that enables person with Alzheimer’s to choose preferred music from different music options.
Kwak et al. (2020); USA	Person with dementia, *n* = 59, G: 46F	Music listening	Receptive	Solo	Yes	Portable media player	Au	Testing	Personally relevant stimulus materials used	BPSD (Beh.)	iPods or digital devices that allowed person with dementia who were long-term residents to listen to personalized playlists.
Orpwood et al. (2010); UK/Canada	Person with dementia (mild-moderate)	Music listening	Receptive	Solo	Yes	Portable media player; Testing	Au	Co-design	Possibility to choose from pre-existing list	Feasibility	a list of technologies, one of which is a simplified music player (it looks like a CD player but is a solid-state mp3 music player) with only an “on” button and an attention-drawing feature that lights up every half hour.
Murphy et al. (2018); USA	Person with dementia, *n* = 17	Music listening	Receptive	Solo	Yes	Portable media player	Au	Testing	Personally relevant stimulus materials used	Feasibility, BPSD (Beh.), QoL	iPod shuffle devices that allowed person with dementia in Assisted living Centres to listen to personalized playlists.
de Kok et al. (2018); Netherlands	Person with dementia, *n* = 7	Music listening	Receptive	Group	Yes	Robot	Au	Testing	Personally relevant stimulus materials used	Feasibility, QoL, interaction (Comm.)	A humanoid robot “pepper” with choreographe software interacted with person with dementia and played songs from their personalized playlist.
Cruz-Sandoval et al. (2018); Mexico/Australia	Person with dementia (MMSE range 9–21, *M* = 15.67, *SD.* = 4.5), *n* = 6; A: Range 71–85 (*M* = 81.5, *SD* = 4.92); G: 5F	Music listening and other activities	Receptive	Group	Yes	Robot	Au	Testing	n/a	Feasibility, BPSD (Beh.)	A conversational humanoid robot “eva” talked and played music for person with dementia to encourage them to socially accept it.
Hodge et al. (2018); UK/Sweden	Person with dementia (1 participant classified as ‘mild diagnosis of dementia’) w/family carers, *n* = 7 (4 PERSON WITH DEMENTIA and 3 family carers - i.e., 3 dyads + 1 participant living on their own); A: Range 51–84; G: 4F	Music listening and watching a concert	Receptive	Dyad	Yes	Virtual Reality	AV	Co-design	Personally relevant stimulus materials used	Feasibility, interaction (explor.)	Exposure to virtual reality environment (through either a head-mounted display or hand-held google cardboard display) to get initial reactions from aesthetically engaging and pleasing experience.
Cheng et al. (2019); Korea	Person with dementia (diagnosed by neurologist), *n* = 7; A: Range 62–82 (*M* = 74, *SD* = 5.5), G: 5F	Music-making	Active	Solo	Yes	Custom device	AV	Co-design	n/a	BPSD (Beh.)	Multi-coloured electronic percussion or keyboard device for person with mild cognitive impairment to play songs in synchrony with presentation displayed on computer screen.
Kenning et al. (2019); Netherlands	Person with dementia, *n* = 11	Music-making	Active	Solo	Yes	Custom device	Au	Co-design	n/a	Feasibility	AirSticks are game controllers that convert gestural movements into sounds to allow for free improvisation.
Houben, Lehn et al. (2020); Netherlands	Person with dementia (moderate or late-stage), *n* = 7; G: 5F; caregivers, *n* = 6; G:6F; forming 7 pairs	Music-making	Active	Dyad	Yes	Custom device	Au	Testing	Possibility to choose from pre-existing list	Feasibility, interaction (Conn.)	Wooden cylinder is covered with two rotatable fabric shells (green and grey), which are twisted by person with dementia and carer together to promote collaborative music making. Sounds at this stage are created through ‘Wizard of Oz’ technique to simulate the prospective instrument’s features.
Cheng and Lee (2018); South Korea	Mild cognitive impairment (not confirmed), *n* = 7; A: Range 64–92 (*M* = 76.9, *SD* = 6.92); G: 4F	Music-making	Active	Solo	Yes	Custom device	AV	Testing	n/a	Feasibility, BPSD (Beh.)	Coloured squares on cardboard are tapped in synchrony with lyrics and colours presented on screen to test difficulty and potential ergonomic design prior to construction
Favilla and Pedell (2013); Australia	Person with dementia and their carers, *n* = 12–14	Music-making	Active	Solo and group	Yes	Application for tablet	Au	Co-design	Possibility to choose from pre-existing list	Feasibility	Transformation of either electronic or keyboard sounds by moving finger along xy axis or by touching buttons on tablet touchscreen (iPad using TouchOSC and interactive music software max)
Favilla and Pedell (2014); Australia	Person with dementia and their carers, *n* = 12–14	Music-making	Active	Solo and group	Yes	Application for tablet	Au	Co-design and testing	Possibility to choose from pre-existing list	Feasibility	Transformation of either electronic or keyboard sounds by moving finger along xy axis or by touching buttons on tablet touchscreen (iPad using TouchOSC and interactive music software max)
Han et al. (2020); Korea	Mild cognitive impairment, *n* = 24; G: 11F	Music-making	Active	Solo and group	Yes	Computer/laptop (w/software, speakers, camera)	AV	Testing	n/a	BPSD (cog.)	Multi-coloured electronic percussion or keyboard device for person with mild cognitive impairment to play in synchrony with presentation displayed on computer screen.
Benveniste et al. (2012); France	Suspicion of Alzheimer’s (MMS score = 10–25), *n* = 9;	Music making - improvisation and following	Active	Solo w/group, Solo	Yes	Video game console	Au	Co-design and testing	Possibility to choose from pre-existing list	BPSD (Beh.), QoL	Nintendo Wii game console and Wiimote controller or Wii pistol that lets people improvise or play chosen songs by pointing at an onscreen virtual keyboard.
Boulay et al. (2011); France	Person with Alzheimer’s (NINCDS-ADRDA criteria; MMS score range 12/30–22/30, *M* = 16.71), *n* = 7; A: 77–94 (*M* = 88.5), G: 4F	Music-making	Active	Solo	Yes	Video game console	Au	Testing	n/a	BPSD (Beh.), QoL	Nintendo Wii game console and Wiimote controller or Wii pistol that lets people play chosen song by pointing at an onscreen virtual keyboard.
Rosseland and Culén (2016); Norway	Person with Alzheimer’s (early stage), *n* = 6 in 3 pairs	Music listening driven by movement	Active	Dyad	Yes	Custom device	AV	Testing	Personally relevant stimulus materials used	Feasibility	Kinect sensor linked to a music player to automatically adjust tempo of music to match the movements of the person with Alzheimer’s.
Morrissey et al. (2016); Australia	Person with dementia, *n* = 25–30	Moving with music	Active	Group	Yes	Video game console	Au	Testing	Possibility to choose from pre-existing list	Feasibility, interaction (Conn.)	SwaytheBand software played known songs and encouraged person with dementia to move to the music by having the top of their PlayStation move controllers light up in synchrony with the beat of the music. Participants were also encouraged to move props in time with music.
Manca et al. (2021); Italy	Person with mild cognitive impairment, *n* = 14;	Music quiz (serious games)	Receptive	Solo w/group	Yes	Application for tablet and Robot	Au	Testing	n/a	Feasibility, BPSD (cog.), QoL	Humanoid robot and tablet that allow person with mild cognitive impairment to play musical memory games.
Capstick (2011); UK	Person with dementia, *n* = 2; G: 2F	Film-making	Active	Dyad	Yes	Computer/laptop (w/software, speakers, camera)	AV	Testing	Personally relevant stimulus materials used	Feasibility	Video editing and narration with mini-camcorder (flip video - size of a digital camera with a large red button for record/stop). Flickr, YouTube and google were also used to find images to be included in the film narratives.
Capstick and Ludwin (2015); UK	Person with Alzheimer’s, *n* = 10	Film-making	Active	Solo	Yes	Computer/laptop (w/software, camera)	AV	Testing	Personally relevant stimulus materials used	Feasibility	Free software was used to put together images into a slideshow of places from the person with Alzheimer’s youth. This was then developed into a film narrative with commentary provided by the person with Alzheimer’s.
Park et al. (2017); Canada	Person with dementia (early stage), *n* = 7; G: 4M	Storytelling	Active	Solo and group	Yes	Computer/laptop (w/software, speakers, camera)	AV	Testing	Personally relevant stimulus materials used	QoL, interaction (Comm., Conn.)	Computers with video editing software (WeVideo) were used for person with dementia to create digital stories.
Stenhouse et al. (2013); UK	Person with dementia (early stage), *n* = 7; Carer, *n* = 1	Storytelling	Active	Solo and group	Yes	Computer/laptop (w/software, speakers, camera)	AV	Co-design	Personally relevant stimulus materials used	QoL, interaction (Conn., explor.)	Laptop and video editing software for person with dementia to create multimedia stories.
Critten and Kucirkova (2019); UK	Person with dementia (mild to moderate), *n* = 3 (+1 spouse); A: Range 72–94 years; G: 1F	Storytelling	Active	Solo w/group	Yes	Application for tablet and smartphone	AV	Testing	Personally relevant stimulus materials used	QoL	Use of pre-existing OurStory app for tablets and smartphones, camera for video and picture-taking, audio recorder, text-input functions, and internet access to create multimedia stories of person with dementia.
Abrahão et al. (2018); Brazil	Person with dementia, *n* = 1; A: 60 years; G: 1F; carers (family members and caregivers), *n* = 4	Storytelling	Active	Group	Yes	Application for smartphone	AV	Testing	Personally relevant stimulus materials used	Feasibility, interaction (Comm.)	Mobile application (com-phone Story maker app for androids) that captures aspects of person with dementia’s everyday life to become a collection of multimedia stories.
Czech et al. (2020); Japan	Person with dementia (moderate-late stage), *n* = 2; Age: 78, 91 years, G: 2F	Storytelling	Active	Solo	Yes	Custom device	AV	Testing	Personally relevant stimulus materials used	Feasibility	Multimodal story album using the TECHTILE toolkit which is a device that enables the design of tactile feeling by recording, editing, playback of the tactile perception of material properties.
Iacono and Marti (2016); Italy	Person with dementia (various levels; MMSE range 13.5–24.7), *n* = 6; A: Range 72–86 (*M* = 79.67)	Storytelling	Active	Group	Yes	Robot	V	Testing	Personally relevant stimulus materials used	Feasibility, BPSD (Beh., cog.)	Robotic seal and interactive toy seal were compared against one another to determine their ability to stimulate person with dementia to share stories or memories.
Jamin et al. (2018); Netherlands	Phase 1, session 1: Various professionals, carers and representatives, *n* = 10; phase 1, session 2: Nursing home residents, *n* = 3; phase 2: Design team, phase 3: Person with dementia, *n* = 10;	Art - interactive	Mixed	Solo and group	Yes	Custom device	V	Co-design	Possibility to choose from pre-existing list	Feasibility, interaction (explor.)	Interactive installation with two touch screens mounted on a wall, a computer, a Kinect sensor (for adapting the perspective to the user’s position) and a roller blind with a string. There are several different scenes available which can be changed by pulling on the string of the blinds.
Luyten et al. (2018); Netherlands	Person with dementia, *n* = 10; G: 8F; care providers, *n* = 1–2; G: 1–2F	Art - interactive	Mixed	Solo and group	Yes	Custom device	V	Testing	Possibility to choose from pre-existing list	Interaction (Comm., explor.)	Interactive installation with two touch screens mounted on a wall, a computer, a Kinect sensor (for adapting the perspective to the user’s position) and a roller blind with a string. There are several different scenes available which can be changed by pulling on the string of the blinds.
Leuty et al. (2013); Canada	Person with dementia (mild to moderate, MMSE score *M* = 16.5, range 15–25), *n* = 6; art therapists, *n* = 6 in dyads	Art - painting	Active	Solo	Yes	Custom device	V	Co-design	Possibility to choose from pre-existing list	Feasibility, interaction (explor.)	ePAD (engaging platform for art development) is an artificial intelligent touch-screen device that estimates person with dementia’s level of engagement, providing prompts when they become disengaged.
Chauhan (2020); UK	Person with dementia, *n* = 7; A: Range 71–100; G: 3F	Art - sculpture-making	Active	Group	Yes	Custom device	V	Testing	Personally relevant stimulus materials used	QoL, interaction (explor.)	1. 3Doodler pen, a handheld 3D printing pen with a heated nozzle that extrudes plastic filaments; 2. touchscreen android tablet using 3D modelling apps (TrueSculpt and Qubism); 3. virtual models using 3D modelling software autodesk maya and cube 3D printer. These were used to develop time-lapse videos and 3D printed physical sculptures.
Lazar et al. (2017a); USA	Person with dementia (vascular; advanced), *n* = 2; G: 2F	Art - creation	Active	Solo	Yes	Custom device	AV	Testing	Personally relevant stimulus materials used	Feasibility	Art installations involving go-pro camera and HP sprout computer (that has vertical touchscreen and projector/camera/scanner above horizontal ‘touchmat’ display) allows visitors to see how artists with dementia interact with their artworks.
Lazar et al. (2017b); USA	Person with dementia, *n* = 2	Art - sharing	Mixed	Solo	Yes	Custom device	V	Co-design (art therapist) and testing (person with dementia)	Personally relevant stimulus materials used	Feasibility	Tablet computer mounted inside a wooden art frame, with 5 clearly labelled physical buttons (arduino board and breakout PCB boards with button pads), and a mirror redirects the tablet camera to capture the space on the table in front of the tablet. They were designed to better enable person with dementia to share their art with others.
Tyack et al. (2017); UK	Person with dementia, *n* = 12; A: Range 64–90 (*M* = 75), G: 4F; informal carers, *n* = 12; A: Range 48–77 (*M* = 66); G:10F	Art - consumption	Receptive	Solo	Yes	Application for tablet	V	Testing	Possibility to choose from pre-existing list	Feasibility, BPSD (Beh., cog.), QoL, interaction (Comm., Conn.)	Touchscreen tablet device (android) for person with dementia to view images of visual art.
Treadaway and Kenning (2015); UK	Study 1: Person with dementia, *n* = 3, families of person with dementia, carers; Study 2: Various professionals, carers and representatives, *n* = 24–30	Object - textiles	Receptive	Solo		Custom device	Au	Co-design	n/a	Feasibility	Different technology items embedded into fabric of garments and blanket; an mp3 player, a textile cat that purrs when it is touched, other sensors that emit light and sounds when touched
Treadaway and Kenning (2016); UK	Study 1: Person with dementia (late-stage), *n* = 3; G: 2F; Study 2: Person with dementia (late-stage), *n* = 7	Objects - textiles	Receptive	Solo		Custom device	Au	Co-design	Personally relevant stimulus materials used	Feasibility, interaction (Comm., Conn., explor.)	Different technology items embedded into fabric of garments and blanket; an mp3 player, a textile cat that purrs when it is touched, other sensors that emit light and sounds when touched
Treadaway et al. (2019); UK	Step 2: Person with dementia (early stage) *n* = 8; dementia experts *n* = 5, caregivers *n* = 11; Step 3: Person with dementia (advanced) *n* = 7; family and caregivers, *n* = 15	Objects	Mixed	Solo		Custom device	AV	Co-design and testing	Possibility to upload personally relevant stimulus materials	Feasibility	1. Soft furry object with weighted arms and legs that wrap around body to simulate hug. A teensy microprocessor, amplifier, miniature speakers, and vibration motor allowed generation of music and a heartbeat upon movement. 2. Steering wheel with haptic feedback, ‘radio’ with preferred songs, and turn signals; all generated through use of teensy 3. Fidget jewellery 4. Giggle balls with small microcontroller that emits sound of laughter when moved.
Golden et al. (2017); USA	Person with dementia, *n* = 27; A: *M* = 79.8 ± 11.4, G: 18F; caregivers, *n* = 27; A: *M* = 53.3 +/13.1, G: 26F	Creative activities (dancing, music, poetry, painting/collage, singing)	Active	Dyad w/carer	Yes	Web application	AV	Testing	n/a	Feasibility	Online platform with videos that instruct family caregivers how to do creative activities with related person with dementia.
Lancioni et al. (2017); Italy	Person with Alzheimer’s (mild-moderate, MM scores 14–22), *n* = 11; A: Range 70–96 years; G: 5F; various professional staff, *n* = 22	Entertainment (music, comedy, films, TV shows)	Receptive	Solo	Yes	Computer/laptop (w/software, speakers, camera)	AV	Testing	Possibility to choose from pre-existing list	Feasibility, BPSD (Beh.), QoL	A laptop computer with an amplifier, a microswitch (handheld pressure device), and software that enables person with Alzheimer’s to independently choose preferred music, comedy, films or tv shows from different options.
Gu et al. (2013); Netherlands	Person with dementia	Light and sound	Receptive	Solo w/group		Custom device	AV	Co-design	n/a	Feasibility	Interactive lighting and sound art installation that provides calm atmosphere and sensory stimulation for improvement of mood.
Azman et al. (2017); Japan	Mild cognitive impairment, *n* = 9; A: Range 65–80 (*M* = 73.6 ± 1.8 years), G: 6F	Dance	Active	Solo		Custom device	AV	Testing	n/a	BPSD (cog.)	Dance video game (StepMania) and dance mat with buttons that are pressed by user’s feet in time with music.

## Results

**Figure 2.**
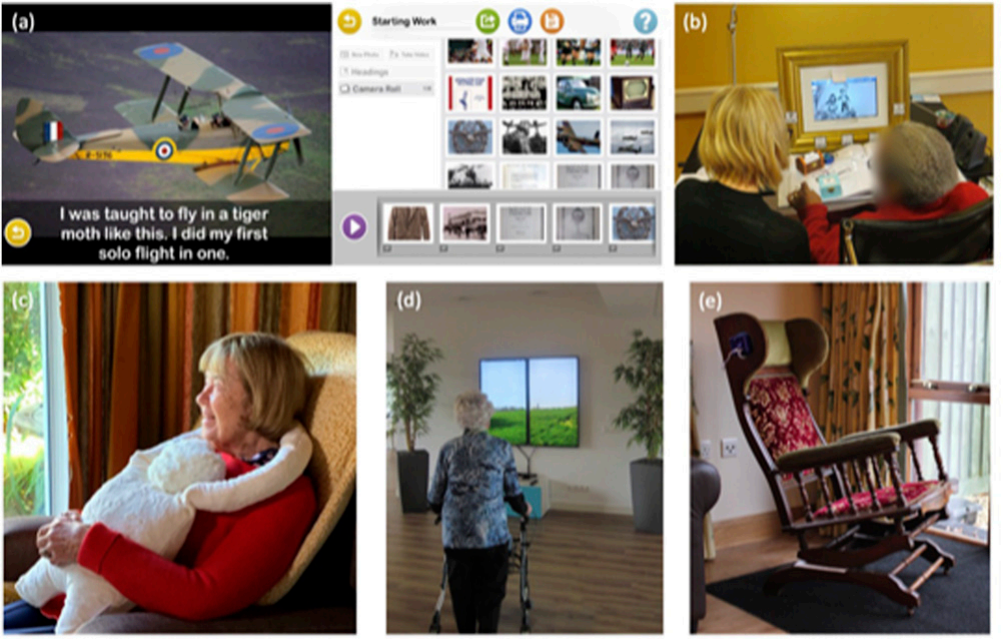
Images from a selection of technologies described in Table 2: (a) two images from the OurStory storytelling application, usage described in Critten & Kucirkova, 2019; (b) art frame described in Lazar et al., 2017b; (c) HUG object described in Treadaway et al., 2019; (d) VENSTER interactive artwork described in Luyten et al., 2018 and Jamin et al., 2018; (e) Rocking chair for music listening described in Bennett et al., 2016. All images reproduced with permission from original authors.

The databases returned 2063 records after duplicate removal and removal for other reasons (e.g. publication year). Fifty-one publications were included in the review (see Figure 1). The main data from the publications are presented in Table 2, with images of a selection of the described technologies in Figure 2.

### Description of the included publications

Of the total 51 publications that were included in the scoping review, 27 were journal articles, 20 were conference proceedings, and four were conference proceedings published as book chapters. The majority of the publications originated from the UK (*n* = 13), followed by the Netherlands (*n* = 9), USA (*n* = 8), Italy (*n* = 5), Australia (*n* = 5), South Korea (*n* = 3), Canada (*n* = 3), Japan (*n* = 2), New Zealand (*n* = 2), France (*n* = 2), Taiwan (*n* = 1), Norway (*n* = 1), Sweden (*n* = 1), Mexico (*n* = 1), and Brazil (*n* = 1).^3^

### Description of art forms

In all 51 papers, an art form was used, whether to promote engagement with the particular art form as a leisure activity, or as one of a list of activities to choose from. Table 2 presents an overview of the main characteristics of the studies. Just over half of the papers focussed on music (*n* = 28) as either the only activity being studied, or the only creative-arts activity studied within the context of a range of alternative non arts-based activities. Three additional studies included music among other activities. Technology was designed for music listening (*n* = 16) (Bennett et al., 2016; Cruz-Sandoval et al., 2018; Cunningham et al., 2019; De Kok et al., 2018; Hodge et al., 2018; Houben, Brankaert, et al., 2020; Hsu et al., 2019; Kwak et al., 2020; Lancioni et al., 2014; Lancioni et al., 2015; Murphy et al., 2018; Nayer et al., 2014; Orpwood et al., 2010; Peeters et al., 2016; Seymour et al., 2017; Thoolen et al., 2019), interactive music listening involving the participant’s active movement (*n* = 2) (Morrissey et al., 2016; Rosseland & Culén, 2016) and music making (*n* = 9) (Benveniste et al., 2012; Boulay et al., 2011; Cheng et al., 2019; Cheng & Lee, 2018; Favilla & Pedell, 2013, 2014; Han et al., 2020; Houben, Lehn, et al., 2020; Kenning et al., 2019). Music-making included versions of familiar instruments such as the keyboard/percussion, or new instruments manipulating sounds. Music-making papers represented tasks that involved reproducing melodies as well as improvisation. There was also one music quiz (*n* = 1) (Manca et al., 2021).

Seven papers reported on technology for visual art. These enabled artwork sharing (*n* = 1) (Lazar et al., 2017b) and watching (*n* = 1) (Tyack et al., 2017), interacting with an artwork (*n* = 2, which report the same device) (Jamin et al., 2018; Luyten et al., 2018) and watching an artist with dementia interact with their work (*n* = 1) (Lazar et al., 2017a). One paper each involved making art using a painting ePad (*n* = 1) (Leuty et al., 2013), and a 3D design interface for objects that can be printed (*n* = 1) (Chauhan, 2020).

The next largest category related to storytelling (*n* = 6) (Abrahão et al., 2018; Critten & Kucirkova, 2019; Czech et al., 2020; Iacono & Marti, 2016; Park et al., 2017; Stenhouse et al., 2013). These applications supported multimedia storytelling including life-stories. The film-making applications (*n* = 2) (Capstick, 2011; Capstick & Ludwin, 2015) had a similar objective of facilitating multimodal narrative, in this case the combination of still or moving images with personal narrative.

Three papers reported on multisensory objects. Two of these were from the same project concerning the design of various textiles with embedded electronics (Treadaway & Kenning, 2015, 2016), and the third explored a tactile object with an embedded music player (Treadaway et al., 2019).

Three papers reported on a variety of creative activities either through online video demonstrations (Golden et al., 2017), access to various multimedia apps (Gilson et al., 2019), or the choice of playing back different media (Lancioni et al., 2017). Single papers examined an application offering dance guidance (Azman et al., 2017), and interactive lighting and sound installation (Gu et al., 2013).

### Description of technologies used

A large number of papers discuss the development of a custom device or devices (*n* = 22), while many others use or develop applications for either a tablet (*n* = 9) or computer (*n* = 8). Other papers discuss the use of a portable media player (*n* = 3), video game system (*n* = 3), VR (*n* = 1), or the use of online software (*n* = 1). Although the majority of papers reported user-testing with people living with dementia or cognitive impairment (*n* = 31), a large number (*n* = 20) detailed co-design processes, incorporating the contributions of people living with dementia, carers, and therapists. In terms of how the devices were designed to adapt to individual users, papers discussed different degrees of personalisation, with none being automated at present. Over half of the papers detailed offering some degree of choice to participants from a pre-existing list (*n* = 38),^4^ whether that was a choice of stimuli, choice of activity or instrument, or choice of either sounds or visual stimuli created by their actions. However, among these studies making choice available, considerably less made use of personally relevant stimulus materials (*n* = 18), or gave the option of later adding personally relevant stimulus materials (*n* = 5). Only two devices offered the ability for the physical interface to be adapted to individual needs or abilities in the moment (*n* = 2). The use of technology for each broad category of arts-based activity will now be described separately.

### Description of activities enabled through technology

#### Music (receptive or mixed)

Participants played pre-recorded songs or audio through a number of different devices. These devices were designed with various options for people living with dementia. Examples of accessible interfaces included the “AAMI” music-listening device (Seymour et al., 2017). This offered different switches or physical buttons for the user interface that could be swapped out depending on the needs of the user. Examples of devices building choice and personalisation into the design included the music-listening device by Lancioni and colleagues (Lancioni et al., 2014, 2015) where a handheld pressure device allowed users to choose between menu items. These music/sound-playing devices were used to listen to either a pre-determined list of music tracks, environmental sounds (e.g. the “Vita” sound cushion, Houben, Brankaert, et al., 2020) or offered a platform to access personalised playlists, annotated playlists or music and photo albums. Devices for listening to music were often used to accompany and support daily tasks, or to improve mood. Feedback from participants was facilitated, including through taking physiological measurements, voice recognition, or ease of control (e.g. the “Resonant Interface Rocking Chair” reported in Bennett et al., 2016 stopped playing music when it was not being rocked). Music (receptive/mixed) as a category made most usage of physical robots (*n* = 2), and was the only art form that made use of portable media (*n* = 3) and virtual reality (*n* = 1). The virtual reality device was designed so the user could access the experience of attending a concert hall with their favourite singer appearing onstage (Hodge et al., 2018).

#### Music (active)

Active engagement with music was promoted by devices which prompted participants to move along with the music. For instance, “SwaytheBand” played through a video games console, made a sequence of gentle light flashes in time with familiar music and people living with dementia were encouraged to move props or controllers at the same time (Morrissey et al., 2016). Other systems monitored the user’s movements (e.g. through a Kinect device) which adjusted the tempo of the familiar song being played (Rosseland & Culén, 2016). Active production of music, most typically designed as playing a musical instrument, was facilitated in various ways. Systems could present visual prompts on screen for a user to press a key/button of associated colour on a device (Cheng & Lee, 2018; Han et al., 2020). For a different example, users were free to create movements which would then be sonified (e.g. using “AirSticks” which convert movements into sounds, Kenning et al., 2019). Other custom devices included a wooden cylinder covered in two separate pieces of fabric that could be rotated by two separate users in an effort to play sounds together (Houben, Lehn, et al., 2020). Active music engagement was the only art form that made use of video game consoles (*n* = 2), and the incidence of co-design is also slightly above average for these studies (*n* = 5 from 11 publications).

#### Storytelling and film-making

In storytelling pre-existing or designed applications supported the creation of multimodal stories using images, audio and video material, related to participants’ daily life or life histories. In some instances, applications such as a robot seal or simulations of tactile objects from the participants’ past served as a prompt to tell stories. In film-making existing technology was used to create multimedia stories using images, recorded speech and other multimedia input. For both of these art forms, a custom device was much less likely to be built, with the majority of the papers describing the use of a pre-existing application for either computer or tablet. Because these projects were less likely to develop their own device, instead using pre-existing applications for tablet or computer, they were also much less likely to involve co-design.

#### Art

Devices promoted engagement with art by enabling control of visual perspectives and by facilitating sharing of art, art consumption, and showing an artist living with dementia at work. For instance, “Venster” (Jamin et al., 2018; Luyten et al., 2018) was a video screen set up to look like a window into a virtual environment, with the blinds functioning as a way to change the scene out of the window. The content in “Venster” often played a major role by inviting and affording certain types of interactions over others, with interactions differing depending on whether the content was calming, activating, or interactive. Other art devices enabled digital art creation in 2D (painting) and 3D (sculpture-making). In Chauhan (2020), participants made virtual and digital sculptures using tablet devices and a 3Doodler pen that extruded heated plastic to make three dimensional objects. Lazar et al. (2017b) developed an art frame with physical buttons that helped to support feelings of agency in the users, improving the sharing process by making it easier to initiate and end messages. The vast majority of these devices were developed as custom devices, which also slightly increased the amount of co-design in these projects.

#### Objects

Creative engagement with multisensory objects was facilitated by using sensors, diverse materials, and possibilities to interact (e.g. modulating sounds or lights). All of these studies completely custom developed the objects through the use of co-design. The LAUGH project (Treadaway et al., 2019) worked to co-design objects that stimulate ‘playful play’ and weren’t goal oriented or reliant on the user’s memory skills. This included the hug device, which was a soft plush doll that wrapped around a user’s shoulders, which played either the sound of a heartbeat or music and was developed to simulate the feeling of a hug. They also developed a steering wheel that vibrated, had working indicator signals and a car radio loaded with recorded personalised music.

#### Miscellaneous

Creative activities or entertainment consumption were facilitated using a digital interface. In some cases, physical objects were used (e.g. mat for instruction of dance steps), or light and sound to modulate the room atmosphere.

### Participants

The target group of the large majority of papers was people living with dementia (*n* = 42) or Alzheimer’s dementia (*n* = 3). In one instance, there was no official diagnosis, but a suspicion of Alzheimer’s based on the MMSE score (mini-mental state exam). The target group of the remaining five papers were people with cognitive impairment. The severity or stage of impairment was not always reported. When reported (total *n* = 23), the condition was reported to be mild or early stage (*n* = 7), mild-to-moderate (*n* = 8), or moderate (*n* = 2). A few studies included various or early and late/advanced stages (*n* = 2), moderate-to-late stages (*n* = 2), or focussed on advanced/late stage (*n* = 2). The median number of people living with dementia, Alzheimer’s dementia, or mild cognitive impairment involved in the studies presented in the papers was 7. Of the studies that reported sample size (total *N* = 48), a minority of studies were case studies with *N* = <3, (*n* = 8), or involved a large number of participants (*n* = 2 with 59 and potentially over 1000 participants). The majority of studies involved either small groups of participants *N* = 4–7 (*n* = 18), or a moderately large number of participants, *N* = 8–27 (*n* = 20). Group size and art form were not closely associated, except that storytelling generally included only a small number of participants (max 7), possibly due to its individualised nature.

### Social context

The majority of activities were oriented towards individual participation (Solo), as reported in *n* = 25 papers. Additionally, applications were for individual participation but in the presence or context of a group (*n* = 6), such that when one individual participated the others in the group watched that person’s activity. Other applications had the flexibility to involve an individual or a group of participants (*n* = 9). A smaller number of studies always involved two (*n* = 5) or a larger group of participants (*n* = 6). This division is however complicated by the fact that activities oriented towards individuals may still in practice involve participation of others, such as carers or therapists, whilst in some of the group activities, individuals take turns doing the activity, such as interacting with a robot to stimulate the sharing of a story. Some of the activities were relatively more frequently reported to be oriented towards solo or group participation. In particular, music listening was relatively often individual, although it can also be done as part of a group and was indeed in a few instances conceived as a group activity. Music making was quite evenly done individually or as part of a group, as was art sharing and creation. Objects were designed for individual interaction, whilst most of the storytelling was done in the context of a group. The vast majority of papers reported the presence of a facilitator to enable the activity (*n* = 43). Three papers reported activities carried out without a facilitator and all of these concerned music listening. Five papers did not report whether a facilitator was present or not. It may be that some art forms initially require a facilitator but participation may then be done without a facilitator, such as interaction with an object or installation. However, the presence of a facilitator when using technology may be important to maintain opportunities for social contact for the person living with dementia (Lazar et al., 2018).

### Outcome measures

Supplementary to the data presented in Table 2, we explored the various types of outcome measures that had been used across the 51 papers included in this review. The papers can be largely classified as having four types of outcome measures. This classification of outcome measures draws upon those advocated for by Dixon and Lazar (2020), and is supported by thematic analyses done by Tyack and Camic (2017) and Treadaway and Kenning (2016). They include: intervention feasibility, behavioural and psychological symptoms (BPSD), quality of life (QoL), and participants’ interactions with their environment. First, more than half of the papers in this review recorded findings on the feasibility of their intervention (*n* = 36) (Abrahão et al., 2018; Bennett et al., 2016; Capstick, 2011; Capstick & Ludwin, 2015; Cheng & Lee, 2018; Cruz-Sandoval et al., 2018; Czech et al., 2020; De Kok et al., 2018; Favilla & Pedell, 2013, 2014; Golden et al., 2017; Gu et al., 2013; Hodge et al., 2018; Houben, Brankaert, et al., 2020; Houben, Lehn, et al., 2020; Hsu et al., 2019; Iacono & Marti, 2016; Jamin et al., 2018; Kenning et al., 2019; Lancioni et al., 2017; Lazar et al., 2017a, 2017b; Leuty et al., 2013; Luyten et al., 2018; Manca et al., 2021; Morrissey et al., 2016; Murphy et al., 2018; Nayer et al., 2014; Orpwood et al., 2010; Peeters et al., 2016; Rosseland & Culén, 2016; Seymour et al., 2017; Thoolen et al., 2019; Treadaway et al., 2019; Treadaway & Kenning, 2015, 2016; Tyack et al., 2017). This was largely reported as whether the activity/device had been successful/unsuccessful, although certain papers reported the iterative steps taken (in terms of pointers for design) to achieve success. Second, 16 of the studies relayed findings related to improvements in their participants’ BPSD. Thirteen of which used behavioural measures,^5^ number of negative behaviours observed, social validation check, task-related error rate over time, number of actions or utterances made when engaging with device; (Benveniste et al., 2012; Boulay et al., 2011; Cheng et al., 2019; Cheng & Lee, 2018; Cruz-Sandoval et al., 2018; Cunningham et al., 2019; Kwak et al., 2020; Lancioni et al., 2014, 2015, 2017; Manca et al., 2021; Murphy et al., 2018; Tyack et al., 2017) to indicate these changes in BPSD, two used cognitive measures^6^ (Azman et al., 2017; Han et al., 2020), while one other used a combination of cognitive and behavioural measures^7^ (Iacono & Marti, 2016). Almost all studies presenting behavioural evidence quantitatively described improvements as occurring along their behavioural measures, with reporting mixed on the types of statistics used, and whether corrections for multiple comparisons were employed. Third, eight studies recorded findings on the changes to the QoL of their participants due to the intervention^8^ (Benveniste et al., 2012; Chauhan, 2020; Cunningham et al., 2019; De Kok et al., 2018; Gilson et al., 2019; Lancioni et al., 2017; Stenhouse et al., 2013; Tyack et al., 2017). QoL was mainly measured quantitatively through the use of questionnaires, but wellbeing was a major theme that came up in a couple of qualitative analyses. Fourth, 14 studies described an increase in participants’ interactions with their environment due to the intervention. Three of these studies indicated an increase in communication (Abrahão et al., 2018; De Kok et al., 2018; Peeters et al., 2016), two an increase in connection between participants and others (Houben, Lehn, et al., 2020; Morrissey et al., 2016), four an increase in exploration (Chauhan, 2020; Hodge et al., 2018; Jamin et al., 2018; Leuty et al., 2013), while five other studies describe these changes as occurring across multiple measures (Luyten et al., 2018; Park et al., 2017; Stenhouse et al., 2013; C Treadaway & Kenning, 2016; Tyack et al., 2017). These studies largely describe their results qualitatively and as post-hoc observations hence there may be a positive bias in reporting “successful” instances.

## Discussion

This review aimed to describe the arts activities supported by developments in technology for older adults living with mild cognitive impairment or dementia. The 51 articles presented demonstrate that technology is being used to enhance a range of creative arts activities for people living with dementia including music, storytelling, and visual arts. Through personal communication, as a result of emailing each corresponding author listed for the articles in Table 2, we are aware of further developments in a number of the different prototype tools presented. A number are now commercially available products (two describing the same interactive artwork device, VENSTER (Jamin et al., 2018; Luyten et al., 2018), the music player from (Orpwood et al., 2010) and HUG, the textile object (Treadaway et al., 2019)), while others are continuing their development (Critten & Kucirkova, 2019; Cunningham et al., 2019; Manca et al., 2021). It is not expected that all devices were designed with the aim of commercialization, and instead could have had a research product as the intended goal. However, this suggests that there is definitely an appetite for technology to support arts engagement with this population, particularly with the knowledge that some of these products have been developed to the point of being commercially available.

Taking the emerging support for technology-supported arts activities for people living with dementia, in combination with the acknowledgement that this population have varied abilities, experiences and interests (Tsekleves, 2021), it may be surprising that most papers typically report no more than one arts activity (be it music, or storytelling or another art-form) and only four papers describe music as part of a list of activities. Only two papers offered a list of different arts-based activities to allow for the diversity of individual interests of older adults living with dementia (Lazar et al, 2018). The implications are that we may be missing important knowledge by examining arts activities (and the technologies developed) in isolation. Comparisons across the arts could be a useful future direction for research. For example, one particular focus arising from the results is for devices that enhance music-based activities including selecting, listening to, and making music, with relatively little comparative activity for visual based arts activities such as selecting and viewing virtual museum/arts exhibits (although there are examples of this outside the scientific literature such as the National Museums Liverpool’s House of Memories app (described in Joddrell & Smith, 2019). A distinct example from the literature on arts-based activities involves the digital sharing of art - this is something yet to be reflected in the music technology research, despite recent anecdotal reports of increased digital music/art sharing as a result of groups meeting online/remotely during the pandemic (Cutler, 2020). Different opportunities for sharing could also be further examined in the music listening context, where the majority of papers reported focusing on solo activity. Here, sharing with a group of others would present further opportunities for social contact.

As a sign of the early stage of the field, the reported studies used small sample sizes, and exploratory approaches rather than systematic comparisons across different levels of cognitive impairment or dementia (similar to studies reporting technologies for reminiscence therapy, see Lazar et al., 2014). The lack of detail on users’/co-designers’ past experiences with the arts and/or technology also means that we do not understand how previous experiences may modify the use/design of these devices. In an example of co-design of new digital musical interfaces for older adults in residential care (MacRitchie et al., 2022), previous experience with traditional musical instruments shaped individuals’ expectations of new devices. Although co-design and further involvement of people living with dementia, their carers and other stakeholders were detailed in a large number of papers, this was not as well reflected in the custom-design devices. The implication here, is that although these devices arguably have the potential to be tailored for individuals, there is currently limited consideration of different levels of previous experience, or interest in the activity itself, which may be leading to certain design decisions. Here is where people living with dementia could be involved more in helping to describe the initial need, following through to how the technology might be implemented for various individuals (Nygård et al., 2019; Tsekleves & Keady, 2021). This is especially seen in the relative lack of adaptability offered by the devices, with only a couple of devices being able to be adjusted to individual needs in the moment.

The second aim of the review was to describe the technological devices that have been developed for these purposes. Although a number of studies report the use of commercially available or off-the-shelf devices such as tablets or smartphone interfaces, the majority of papers report custom-made devices. Taking the outcome measure reported as a loose proxy indicator of development of each prototype, the majority of devices report intervention feasibility measures, implying they are either mostly at prototype stage, or they are developed devices (such as smartphones) that are being used for the first time in this type of creative arts intervention. This focus on validating the success of a device/intervention is typical across design research in dementia, where most of the research is at preliminary or pilot-stage (Tsekleves & Keady, 2021). Secondary focus then appears on health outcomes such as behavioural and psychological symptoms and QoL, with the least focus on interactions with the environment, typically post-hoc observations about a range of indicators. In order to progress the field, future research could consider more planned measures into different aspects of interaction during an activity, depending on what is most important for those living with dementia. For example, in the music and dementia literature, in-the-moment experiences are just as valuable to those living with dementia and their carers, and can reflect positive moments of agency, connection and sharing (Dowlen et al., 2021).

A key limitation to this review is that it is restricted to peer-reviewed published research. There may be other types of commercially available technology products that are in use for dementia populations that have not necessarily been reported through the scientific literature. In order to account for this, it would be informative to explore how arts organisations have adapted their digital offerings during the pandemic to people living with dementia.

Despite limitations, the 51 papers detailed in this review offer rich insights on the field of technology-enhanced creative arts activities for older adults living with dementia. The challenge for future research is to move more of these technologies past the prototyping stage and consider how we might design creative tools for a range of interests to enhance the lives of those living with dementia. Feasibility in many instances can be assured. The next important step will be to identify more precisely what characteristics promote and inhibit engagement and enjoyment, comparing across designs, arts activities, populations and longer-term usages. This review has identified some of the parameters that vary across designs and are important to consider including social context, type of design, type of engagement and outcome objectives. For applications in real-life, it may not be about optimising one design, but the option to flexibly switch between modes of engagement and adapt to the social context that may be most powerful.

## Notes

1 Although earlier models of touchscreen technology exist, the launch of the iPad is widely considered as timepoint of re-vamping of the tablet market. See Joddrell and Astell (2016) for a review of how touchscreen technologies have been used with people living with dementia in general and (Hung et al., 2021) for a review of how tablets have been used to support social connectivity in care home settings.

2 The decision to exclude technologies developed for individuals with young onset dementia was because these individuals will likely experience different issues to older adults living with dementia, as well as particularly with maintaining or participating in hobbies and interests (see Alzheimer’s Society https://about-dementia/types-dementia/particular-issues-faced-younger-people-dementia).

3 Five papers were classed as originating from multiple countries.

4 This does not include two papers that described the ability to skip or pause songs (*n* = 2).

5 Examples of standardised behvioural tests used include the Cohen-Mansfield Agitation Inventory (CMAI) and the Neuropsychiatric Inventory - Nursing Home (NPI–NH).

6 Examples of cognitive measures were using functional near-infrared spectroscopy (fNIRS), or tests including the Mini-Mental State Exam (MMSE), Montreal Cognitive Assessment (MOCA) or Clinical Dementia Rating Scale–Sum of Boxes (CDR-SB).

7 Behavioural measures included the number of words spoken and complexity of story. Cognitive measures included the change in MMSE score described.

8 Examples of quality of life measures were the Quality of Life in Alzheimer’s Disease scale (QoL-AD), Self-Assessment Manikin scale, and the visual analog mood scale (VAMS).

## References

[bibr1-14713012221127359] Abrahão A. R. da Silva P. F. C. Frohlich D. M. Chrysanthaki T. Gratão A. Castro P. (2018). Mobile digital storytelling in a Brazilian care home. In Zhou J. Salvendy G. (Eds.), 4th International Conference on Human Aspects of IT for the Aged Population, ITAP 2018 Held as Part of HCI International 2018 (Vol. 10926 LNCS, pp. 403–421). Springer. 10.1007/978-3-319-92034-4_31

[bibr90-14713012221127359] Alm N. Astell A. Gowans G. Dye R. Ellis M. Vaugan P. Newell A. (2007). An Interactive Entertainment System Usable by Elderly People with Dementia. In Stephanidis C. (Ed.), Universal Access in HCI, Part II, HCII 2007, Lecture Notes in Computer Science 4555 (pp. 617–623). Springer Verlag. 10.1007/978-3-319-92034-4_31

[bibr2-14713012221127359] Arksey H. O’Malley L. (2005). Scoping studies: Towards a methodological framework. International Journal of Social Research Methodology: Theory and Practice, 8(1), 19–32. 10.1080/1364557032000119616

[bibr3-14713012221127359] Astell A. J. Bouranis N. Hoey J. Lindauer A. Mihailidis A. Nugent C. Robillard J. M. (2019). Technology and dementia: The future is now. Dementia and Geriatric Cognitive Disorders, 47(3), 131–139. 10.1159/000497800 31247624 PMC6643496

[bibr4-14713012221127359] Azman N. Suzuki K. Suzuki T. Ono Y. Edanaka Y. Kunieda F. Nakata M. Watanabe K. (2017). Effect of dance video game training on elderly’s cognitive function. Transactions of Japanese Society for Medical and Biological Engineering, 55(Proc), 526–529. 10.11239/jsmbe.55Annual.526

[bibr5-14713012221127359] Bennett P. Cater K. Hinder H. (2016). Rekindling imagination in dementia care with the resonant interface rocking Chair. In Proceedings of the 2016 CHI Conference Extended Abstracts on Human Factors in Computing Systems (CHI EA '16), 07–12 May 2016, San Jose, California, USA. 10.1145/2851581.2892505

[bibr6-14713012221127359] Benveniste S. Jouvelot P. Pin B. Péquignot R. (2012). The MINWii project: Renarcissization of patients suffering from Alzheimer’s disease through video game-based music therapy. Entertainment Computing, 3(4), 111–120. 10.1016/j.entcom.2011.12.004

[bibr7-14713012221127359] Boulay M. Benveniste S. Boespflug S. Jouvelot P. Rigaud A. S. (2011). A pilot usability study of MINWii, a music therapy game for demented patients. Technology and Health Care, 19(4), 233–246. 10.3233/THC-2011-0628 21849735

[bibr8-14713012221127359] Brancatisano O. Baird A. Thompson W. F. (2020). Why is music therapeutic for neurological disorders? The therapeutic music capacities model. Neuroscience and Biobehavioral Reviews, 112, 600–615. 10.1016/j.neubiorev.2020.02.008 32050086

[bibr9-14713012221127359] Capstick A. (2011). Travels with a Flipcam: Bringing the community to people with dementia in a day care setting through visual technology. Visual Studies, 26(2), 142–147. 10.1080/1472586X.2011.571890

[bibr10-14713012221127359] Capstick A. Ludwin K. (2015). Place memory and dementia: Findings from participatory film-making in long-term social care. Health & Place, 34, 157–163. 10.1016/j.healthplace.2015.05.012 26046299

[bibr11-14713012221127359] Chauhan S. (2020). Dementia and sculpture-making: Exploring artistic responses of people with dementia. Dementia, 19(2), 416–432. 10.1177/1471301218777446 29783890

[bibr12-14713012221127359] Cheng H. I. Alifa R. Lee H. (2019). The effectiveness of music therapy system for the elderly with mild cognitive impairment. In Proceedings of the 2019 7th International Conference on Information Technology: IoT and Smart City (Shanghai, China, December 20-23, 2019) (ICIT 2019), New York, NY, USA. Association for Computing Machinery, 445–448. 10.1145/3377170.3377270

[bibr13-14713012221127359] Cheng H.-I. Lee H. (2018). A pilot study to develop digital music instrument. In Weghorn H. Isaias P. Rodrigues L. (Eds.), International conferences on WWW/internet, ICWI 2018 and applied computing 2018 (pp. 385–388). IADIS Press. https://www.scopus.com/inward/record.uri?eid=2-s2.0-85060273091&partnerID=40&md5=e5987ea8347bae48f46b87c3d56750f7

[bibr14-14713012221127359] Creech A. (2019). Using music technology creatively to enrich later-life: A literature review. Frontiers in Psychology, 10, 1–14. 10.3389/fpsyg.2019.00117 30761052 PMC6363696

[bibr15-14713012221127359] Critten V. Kucirkova N. (2019). ‘It brings it all back, all those good times; it makes me go close to tears’. Creating digital personalised stories with people who have dementia. Dementia, 18(3), 864–881. 10.1177/1471301217691162 28161989

[bibr16-14713012221127359] Cruz-Sandoval D. Favela J. Sandoval E. B. (2018). Strategies to facilitate the acceptance of a social robot by people with dementia. In Proceedings of the 13th Annual ACM/IEEE International Conference on Human Robot Interaction, March 5–8, 2018, Chicago, IL, USA, 95–96. HRI'18. 10.1145/3173386.3177081

[bibr17-14713012221127359] Cuffaro L. Di Lorenzo F. Bonavita S. Tedeschi G. Leocani L. Lavorgna L. (2020). Dementia care and COVID-19 pandemic: A necessary digital revolution. Neurological Sciences, 41, 1977–1979. 10.1007/s10072-020-04512-4 32556746 PMC7298162

[bibr18-14713012221127359] Cunningham S. Brill M. Whalley J. H. Read R. Anderson G. Edwards S. Picking R. (2019). Assessing wellbeing in people living with dementia using reminiscence music with a mobile app (memory tracks): A mixed methods cohort study. Journal of Healthcare Engineering, 2019, 1–10. 10.1155/2019/8924273 PMC6748176 31583068

[bibr19-14713012221127359] Cutler D. (2020). Key Workers: Creative ageing in lockdown and after. https://baringfoundation.org.uk/resource/key-workers-creative-ageing-in-lockdown-and-after/

[bibr20-14713012221127359] Czech E. Shibasaki M. Tsuchiya K. Peiris R. L. Minamizawa K. (2020). Discovering narratives: Multi-sensory approach towards designing with people with dementia. In Extended Abstracts of the 2020 CHI Conference on Human Factors in Computing Systems, CHI EA 2020, Honolulu, HI, USA, April 25-30, 2020. 10.1145/3334480.3375209

[bibr21-14713012221127359] De Kok R. Rothweiler J. Scholten L. Van Zoest M. Boumans R. Neerincx M. (2018). Combining social robotics and music as a non-medical treatment for people with dementia. In RO-MAN 2018 - 27th IEEE International Symposium on Robot and Human Interactive Communication, Tai’an, China, 27–31 August 2018, pp. 465–467. 10.1109/ROMAN.2018.8525813

[bibr22-14713012221127359] Dingle G. A. Sharman L. S. Bauer Z. Beckman E. Broughton M. Bunzli E. Davidson R. Draper G. Fairley S. Farrell C. Flynn L. M. Gomersall S. Hong M. Larwood J. Lee C. Lee J. Nitschinsk L. Peluso N. Reedman S. E. Wright O. R. L. (2021). How do music activities affect health and well-being? A scoping review of studies examining psychosocial mechanisms. Frontiers in Psychology, 12, 1–12. 10.3389/fpsyg.2021.713818 PMC8455907 34566791

[bibr23-14713012221127359] Dixon E. Lazar A. (2020). Approach matters: Linking practitioner approaches to technology design for people with dementia. In Proceedings of the 2020 CHI Conference on Human Factors in Computing Systems, Honolulu, HI, USA, April 25-30, 2020 (pp. 1–15). 10.1145/3313831.3376432 PMC7383934 32719832

[bibr24-14713012221127359] Dowlen R. Keady J. Milligan C. Swarbrick C. Ponsillo N. Geddes L. Riley B. (2021). In the moment with music: An exploration of the embodied and sensory experiences of people living with dementia during improvised music-making. Ageing and Society, 1–23. 10.1017/S0144686X21000210

[bibr25-14713012221127359] Dowson B. Atkinson R. Barnes J. Barone C. Cutts N. Donnebaum E. Hung Hsu M. Lo Coco I. John G. Meadows G. O’Neill A. Noble D. Norman G. Pfende F. Quinn P. Warren A. Watkins C. Schneider J. (2021). Innovative digital approaches to music-making for people with dementia in response to the COVID-19 pandemic: Current practice and recommendations. Frontiers in Psychology, 12, 1273. 10.3389/fpsyg.2021.625258 PMC8103026 33967893

[bibr26-14713012221127359] Fancourt D. Aughterson H. Finn S. Walker E. Steptoe A. (2021). How leisure activities affect health: A narrative review and multi-level theoretical framework of mechanisms of action. The Lancet Psychiatry, 8(4), 329–329. 10.1016/s2215-0366(20)30384-9 33581775 PMC7613155

[bibr27-14713012221127359] Fancourt D. Steptoe A. Cadar D. (2020). Community engagement and dementia risk: Time-to-event analyses from a national cohort study. Journal of Epidemiology and Community Health, 74, 71–77. 10.1136/jech-2019-213029 31662344 PMC6929705

[bibr28-14713012221127359] Favilla S. Pedell S. (2013). Touch screen ensemble music: Collaborative interaction for older people with dementia. In Proceedings of the 25th Australian Computer-Human Interaction Conference: Augmentation, Application, Innovation, Collaboration (OzCHI’13), Australia, 25–29 November 2013, pp. 481–484. 10.1145/2541016.2541088

[bibr29-14713012221127359] Favilla S. Pedell S. (2014). Touch screen collaborative music: Designing NIME for older people with dementia. In Proceedings of the International Conference on New Interfaces for Musical Expression, New York, NY, 6–10 June 2014, pp. 35–39. http://www.nime.org/proceedings/2014/nime2014_417.pdf

[bibr30-14713012221127359] Garrido S. Dunne L. Perz J. Chang E. Stevens C. J. (2020). The use of music in aged care facilities: A mixed-methods study. Journal of Health Psychology, 25(10–11), 1425–1438. 10.1177/1359105318758861 29468892

[bibr31-14713012221127359] Gilson A. Dodds D. Kaur A. Potteiger M. Ford J. H. (2019). Using computer tablets to improve moods for older adults with dementia and interactions with their caregivers: Pilot intervention study. JMIR Formative Research, 3(3), 1–13. 10.2196/14530 PMC6751094 31482847

[bibr32-14713012221127359] Golden A. Gammonley D. Hanna Powell G. Wan T. T. (2017). The challenges of developing a participatory arts intervention for caregivers of persons with dementia. Cureus, 9(4), e1154. 10.7759/cureus.1154 28503390 PMC5426821

[bibr33-14713012221127359] Gordon-Nesbitt R. Howarth A. , etal. (2020). The arts and the social determinants of health: Findings from an inquiry conducted by the United Kingdom all-party parliamentary group on arts, health and wellbeing. Arts & Health, 12, 1–22. 10.1080/17533015.2019.1567563 31038422

[bibr34-14713012221127359] Gu J. Zhang Y. Hu J. (2013). Lighting and sound installation for elderly with dementia. In Proceedings - 2013 International Conference on Culture and Computing, Culture and Computing, 16–18 September 2013, Kyoto, Japan, pp. 169–170. 10.1109/CultureComputing.2013.50

[bibr35-14713012221127359] Han E. Park J. Kim H. Jo G. Do H. K. Lee B. I. (2020). Cognitive intervention with musical stimuli using digital devices on mild cognitive impairment: A pilot study. Healthcare, 8(1), 45. 10.3390/healthcare8010045 32106559 PMC7151070

[bibr36-14713012221127359] Hodge J. Balaam M. Hastings S. Morrissey K. (2018). Exploring the design of tailored virtual reality experiences for people with dementia. In Proceedings of the 2018 CHI Conference on Human Factors in Computing Systems, CHI 2018, Montreal, QC, 21–26 April 2018. 10.1145/3173574.3174088

[bibr37-14713012221127359] Houben M. Brankaert R. Bakker S. Kenning G. Bongers I. Eggen B. (2020). The role of everyday sounds in advanced dementia care. In Proceedings of the 2020 CHI Conference on Human Factors in Computing Systems, Honolulu, HI, 25–30 April 2020. 10.1145/3313831.3376577

[bibr38-14713012221127359] Houben M. Lehn B. van den Brink N. Diks S. Verhoef J. Brankaert R. (2020). Turnaround: Exploring care relations in dementia through design. In Extended Abstracts of the 2020 CHI Conference on Human Factors in Computing Systems, Honolulu, HI, 25–30 April 2020, pp. 1–8. 10.1145/3334480.3382846

[bibr39-14713012221127359] Hsu W.-Y. Hsieh L.-L. Su Y.-H. Su M.-J. Su L. Chen M.-C. Chan H.-T. (2019). Establishment of a music care system for the elderly in a long-term care facility. In Proceedings of the 7th IEEE International Conference on E-Health and Bioengineering. EHB 2019, Iasi, Romania, 21–23 November 2019. 10.1109/EHB47216.2019.8970095

[bibr91-14713012221127359] Hung L. Chow B. Shadarevian J. O’Neill R. Berndt A. Wallsworth C. Horne N. Gregorio M. Mann J. Son C. Chaudhury H. (2020). Using touchscreen tablets to support social connections and reduce responsive behaviours among people with dementia in care settings: A scoping review. Dementia, 20(3), 1124–1143. 10.1177/1471301220922745 32380856 PMC8044627

[bibr40-14713012221127359] Iacono I. Marti P. (2016). Narratives and emotions in seniors affected by dementia: A comparative study using a robot and a toy. In Proceedings of the 25th IEEE International Symposium on Robot and Human Interactive Communication, RO-MAN 2016, New York, NY, 26–31 August 2016, pp. 318–323. 10.1109/ROMAN.2016.7745149

[bibr41-14713012221127359] Jamin G. Luyten T. Delsing R. Braun S. (2018). The process of co-creating the interface for VENSTER, an interactive artwork for nursing home residents with dementia. Disability and Rehabilitation. Assistive Technology, 13(8), 809–818. 10.1080/17483107.2017.1385102 29037109

[bibr92-14713012221127359] Joddrell P. Astell A. (2016). Studies involving people with dementia and touchscreen technology: A literature review. JMIR Rehabilitation and Assistive Technologies, 3(2). 10.2196/rehab.5788 PMC5454556 28582254

[bibr42-14713012221127359] Joddrell P. Smith S. K. (2019). Leisure activities and technology with dementia. In Astell A. Smith S. K. Joddrell P. (Eds.), Using Technology in dementia care: A Guide to Technology Solutions for everyday living (pp. 105–120). Jessica Kingsley Publishers.

[bibr43-14713012221127359] Kenning G. Ilsar A. Brankaert R. Evans M. (2019). Improvisation and reciprocal design: Soundplay for dementia. In Brankaert R. Ijsselsteijn W. A. (Eds.), Dementia Lab 2019. Making Design Work: Engaging with Dementia in Context. D-Lab 2019. Communications in Computer and Information Science (vol. 1117, pp. 82–91). Springer. 10.1007/978-3-030-33540-3_8

[bibr44-14713012221127359] Klimova B. Valis M. Kuca K. (2018). Exploring assistive technology as a potential beneficial intervention tool for people with alzheimer’s disease – A systematic review. Neuropsychiatric Disease and Treatment, 14, 3151–3158. 10.2147/NDT.S181849 30532546 PMC6247949

[bibr45-14713012221127359] Krause A. E. North A. C. Davidson J. W. (2019). Using self-determination theory to examine musical participation and well-being. Frontiers in Psychology, 10, 1–12. 10.3389/fpsyg.2019.00405 30881330 PMC6407371

[bibr46-14713012221127359] Kwak J. Anderson K. O’Connell Valuch K. (2020). Findings from a prospective randomized controlled trial of an individualized music listening program for persons with dementia. Journal of Applied Gerontology, 39(6), 567–575. 10.1177/0733464818778991 29871544

[bibr47-14713012221127359] Lancioni G. E. O’Reilly M. F. Sigafoos J. D’Amico F. Pinto K. De Vanna F. Scordamaglia A. (2017). Persons with mild and moderate alzheimer’s disease use simple technology to support their leisure engagement. Advances in Neurodevelopmental Disorders, 1, 31–36. 10.1007/s41252-016-0002-4

[bibr48-14713012221127359] Lancioni G. E. Singh N. N. O’Reilly M. F. Sigafoos J. D’Amico F. Sasanelli G. De Vanna F. Signorino M. (2015). Persons with Alzheimer’s disease engage in leisure and mild physical activity with the support of technology-aided programs. Research in Developmental Disabilities, 37, 55–63. 10.1016/j.ridd.2014.11.004 25460220

[bibr49-14713012221127359] Lancioni G. E. Singh N. N. O’Reilly M. F. Sigafoos J. Renna C. Pinto K. De Vanna F. Caffò A. O. Stasolla F. (2014). Persons with moderate Alzheimer’s disease use simple technology aids to manage daily activities and leisure occupation. Research in Developmental Disabilities, 35(9), 2117–2128. 10.1016/j.ridd.2014.05.002 24881006

[bibr50-14713012221127359] Lazar A. Edasis C. Piper A. M. (2017a). A critical lens on dementia and design in HCI. In Proceedings of the 2017 CHI Conference on Human Factors in Computing Systems, CHI 2017, Denver, CO, 6–11 May 2017, pp. 2175–2188. 10.1145/3025453.3025522

[bibr51-14713012221127359] Lazar A. Edasis C. Piper A. M. (2017b). Supporting people with dementia in digital social sharing. In Proceedings of the 2017 CHI Conference on Human Factors in Computing Systems, CHI 2017, Denver, CO, 6–11 May 2017, pp. 2149–2162. 10.1145/3025453.3025586

[bibr52-14713012221127359] Lazar A. Thompson H. Demiris G. (2014). A systematic review of the use of technology for reminiscence therapy HHS public access author manuscript. Health Education & Behavior, 41(1), 51–61. 10.1177/1090198114537067 PMC4844844 25274711

[bibr53-14713012221127359] Lazar A. Thompson H. J. Demiris G. (2018). Design recommendations for recreational systems involving older adults living with dementia. Journal of Applied Gerontology, 37(5), 595–619. 10.1177/0733464816643880 27106883

[bibr54-14713012221127359] Leuty V. Boger J. Young L. Hoey J. Mihailidis A. (2013). Engaging older adults with dementia in creative occupations using artificially intelligent assistive technology. Assistive Technology, 25(2), 72–79. 10.1080/10400435.2012.715113 23923689

[bibr55-14713012221127359] Levac D. Colquhoun H. O’Brien K. K. (2010). Scoping studies: Advancing the methodology. Implementation Science, 5(69), 1–9. 10.1186/1748-5908-5-69 20854677 PMC2954944

[bibr56-14713012221127359] Lourida I. Gwernan-Jones R. Abbott R. Rogers M. Green C. Ball S. Hemsley A. Cheeseman D. Clare L. Moore D. Hussey C. Coxon G. Llewellyn D. J. Naldrett T. Thompson Coon J. (2020). Activity interventions to improve the experience of care in hospital for people living with dementia: A systematic review. BMC Geriatrics, 20, 131. 10.1186/s12877-020-01534-7 32272890 PMC7146899

[bibr57-14713012221127359] Luyten T. Braun S. Jamin G. van Hooren S. de Witte L. (2018). How nursing home residents with dementia respond to the interactive art installation ‘VENSTER’: A pilot study. Disability and Rehabilitation. Assistive Technology, 13(1), 87–94. 10.1080/17483107.2017.1290701 28287047

[bibr93-14713012221127359] MacRitchie J. Breaden M. Taylor J. R. Milne A. J. (2022). Exploring older adult needs and preferences for technology-assisted group music-making. A qualitative analysis of data collected during the participatory user-centred design process. Disability and rehabilitation. Assistive technology, 1–10. 10.1080/17483107.2022.2077461 35658719

[bibr58-14713012221127359] Manca M. Paternò F. Santoro C. Zedda E. Braschi C. Franco R. Sale A. (2021). The impact of serious games with humanoid robots on mild cognitive impairment older adults. International Journal of Human Computer Studies, 145, 102509. 10.1016/j.ijhcs.2020.102509

[bibr59-14713012221127359] Morrissey K. Wood G. Green D. Pantidi N. McCarthy J. (2016). I’m a rambler, I’m a gambler, I’m a long way from home. In Proceedings of the 2016 ACM Conference on Designing Interactive Systems, June 4-8, 2016, Brisbane, QLD, Australia, (pp. 1008–1020). 10.1145/2901790.2901798

[bibr60-14713012221127359] Murphy K. Liu W. W. Goltz D. Fixsen E. Kirchner S. Hu J. White H. (2018). Implementation of personalized music listening for assisted living residents with dementia. Geriatric Nursing, 39(5), 560–565. 10.1016/j.gerinurse.2018.04.001 29731392 PMC6812488

[bibr61-14713012221127359] Nayer K. De Bono A. Coxon S. Van Der Ploeg E. O’Connor D. (2014). Memory box: A personalised multimedia device for individuals with dementia. In Stephanidis C. Antona M. (Eds.), Universal Access in Human-Computer Interaction. Aging and Assistive Environments. UAHCI 2014. Lecture Notes in Computer Science (Vol 8515). Springer Verlag. 10.1007/978-3-319-07446-7_32

[bibr62-14713012221127359] Nygård L. Malinowsky C. Rosenberg L. (2019). Assessing the needs of people with dementia for technology. In Astell A. Smith S. K. Joddrell P. (Eds.), Using technology in dementia care: A guide to technology solutions for everyday living (pp. 27–42). Jessica Kingsley Publishers.

[bibr63-14713012221127359] Orpwood R. Chadd J. Howcroft D. Sixsmith A. Torrington J. Gibson G. Chalfont G. (2010). Designing technology to improve quality of life for people with dementia: User-led approaches. Universal Access in the Information Society, 9, 249–259. 10.1007/s10209-009-0172-1

[bibr64-14713012221127359] Paolantonio P. Pedrazzani C. Cavalli S. Williamon A. (2021). Music in the life of nursing home residents. Arts and Health, 1–17. 10.1080/17533015.2021.1942938 34275413

[bibr65-14713012221127359] Park E. Owens H. Kaufman D. Liu L. (2017). Digital storytelling and dementia. In Zhou J. Salvendy G. (Eds.), Human Aspects of IT for the Aged Population. Applications, Services and Contexts. ITAP 2017, Lecture Notes in Computer Science (Vol. 10298). Springer. 10.1007/978-3-319-58536-9_35

[bibr66-14713012221127359] Peeters M. M. M. Harbers M. Neerincx M. A. (2016). Designing a personal music assistant that enhances the social, cognitive, and affective experiences of people with dementia. Computers in Human Behavior, 63, 727–737. 10.1016/j.chb.2016.06.003

[bibr67-14713012221127359] Peine A. (2019). Technology and ageing—theoretical propositions from science and technology studies (STS). In Ageing and digital technology (pp. 51–64). Springer.

[bibr94-14713012221127359] Riley P. Alm N. Newell A. (2009). An interactive tool to promote musical creativity in people with dementia. Computers in Human Behavior, 25(3), 599–608. 10.1016/j.chb.2008.08.014

[bibr68-14713012221127359] Reilly S. T. Harding A. J. E. Morbey H. Ahmed F. Williamson P. R. Swarbrick C. Leroi I. Davies L. Reeves D. Holland F. Hann M. Keady J. (2020). What is important to people with dementia living at home? A set of core outcome items for use in the evaluation of non-pharmacological community-based health and social care interventions. Age and Ageing, 49(4), 664–671. 10.1093/ageing/afaa015

[bibr69-14713012221127359] Rogers N. T. Fancourt D. (2020). Cultural engagement is a risk-reducing factor for frailty incidence and progression. Journals of Gerontology - Series B Psychological Sciences and Social Sciences, 75(3), 571–576. 10.1093/geronb/gbz004 30624696 PMC7768715

[bibr70-14713012221127359] Rosseland R. B. Culén A. L. (2016). Repmoves: Stories that a rhythmic interaction device for seniors can tell. IADIS International Journal on Computer Science and Information Systems, 11(2), 104–118.

[bibr71-14713012221127359] Seymour P. F. Matejka J. Foulds G. Petelycky I. Anderson F. (2017). AMI: An adaptable music interface to support the varying needs of people with dementia. In Proceedings of the 19th International ACM SIGACCESS Conference on Computers and Accessibility, ASSETS 2017, Baltimore MD, 20 October–1 November 2017, pp. 150–154. 10.1145/3132525

[bibr72-14713012221127359] Stenhouse R. Tait J. Hardy P. Sumner T. (2013). Dangling conversations: Reflections on the process of creating digital stories during a workshop with people with early-stage dementia. Journal of Psychiatric and Mental Health Nursing, 20(2), 134–141. 10.1111/j.1365-2850.2012.01900.x 22413774

[bibr73-14713012221127359] Sweeney L. Clarke C. Wolverson E. (2021). The use of everyday technologies to enhance well-being and enjoyment for people living with dementia: A systematic literature review and narrative synthesis. Dementia, 20(4), 1470–1495. 10.1177/1471301220929534 32539471

[bibr74-14713012221127359] The Commission on Dementia and Music (2018). What would life be - Without a song or a dance, what are we?. A report from the commission on dementia and music. https://ilcuk.org.uk/what-would-life-be-without-a-song-or-dance-what-are-we/

[bibr75-14713012221127359] Thoolen M. Brankaert R. Lu Y. (2019). SENTIC: A tailored interface design for people with dementia to access music. In: DIS 2019 Companion - Companion Publication of the 2019 ACM Designing Interactive Systems Conference, New York, NY, 23–28 June 2019, pp. 57–60. 10.1145/3301019.3325152

[bibr76-14713012221127359] Treadaway C. Kenning G. (2015). Designing sensory textiles for dementia. In Proceedings of The Third International Conference on Design Creativity, Ahmedabad, India, 19–22 January 2015, pp. 1–11. http://hdl.handle.net/10453/122535

[bibr77-14713012221127359] Treadaway C. Kenning G. (2016). Sensor e-textiles: Person centred co-design for people with late stage dementia. Working with Older People, 20(2), 76–85. 10.1108/wwop-09-2015-0022

[bibr78-14713012221127359] Treadaway C. Taylor A. Fennell J. (2019). Compassionate design for dementia care. International Journal of Design Creativity and Innovation, 7(3), 144–157. 10.1080/21650349.2018.1501280

[bibr79-14713012221127359] Tsekleves E. (2021). Things: Design interventions against dementia. In Tsekleves E. Keady J. (Eds.), Design for people living with dementia: Interactions and innovations (pp. 63–89). Routledge.

[bibr80-14713012221127359] Tsekleves E. Keady J. (2021). Future: Challenges & emerging opportunities. In Tsekleves E. Keady J. (Eds.), Design for people living with dementia: Interactions and innovations (pp. 141–157). Routledge.

[bibr81-14713012221127359] Tyack C. Camic P. M. (2017). Touchscreen interventions and the well-being of people with dementia and caregivers: A systematic review. International psychogeriatrics, 29(8), 1261–1280. 10.1017/s1041610217000667 28446258

[bibr82-14713012221127359] Tyack C. Camic P. M. Heron M. J. Hulbert S. (2017). Viewing art on a tablet computer: A well-being intervention for people with dementia and their caregivers. Journal of Applied Gerontology, 36(7), 864–894. 10.1177/0733464815617287 26675353

[bibr83-14713012221127359] Tymoszuk U. Perkins R. Spiro N. Williamon A. Fancourt D. (2020). Longitudinal associations between short-term, repeated, and sustained arts engagement and well-being outcomes in older adults. The Journals of Gerontology. Series B, Psychological Sciences and Social Sciences, 75(7), 1609–1619. 10.1093/geronb/gbz085 31287550 PMC7424284

[bibr84-14713012221127359] van der Steen J. T. van Soest-Poortvliet M. C. van der Wouden J. C. Bruinsma M. S. Scholten R. J. Vink A. C. (2017). Music-based therapeutic interventions for people with dementia. Cochrane Database of Systematic Reviews, 2018(7), Cd003477. 10.1002/14651858.CD003477.pub4 PMC6481517 28462986

[bibr85-14713012221127359] Wang S. Mak H. W. Fancourt D. (2020). Arts, mental distress, mental health functioning & life satisfaction: Fixed-effects analyses of a nationally-representative panel study. BMC Public Health, 20, 1–9. 10.1186/s12889-019-8109-y 32046670 PMC7014626

[bibr86-14713012221127359] Wittenberg R. Hu B. Barraza-Araiza L. F. Rehill A. (2019). Projections of older people with dementia and costs of dementia care in the United Kingdom, 2019–2040. CPEC Working Paper 5, 1–79. https://www.alzheimers.org.uk/sites/default/files/2019-11/cpec_report_november_2019.pdf

[bibr87-14713012221127359] World Health Organization (2018). Assistive technology. https://www.who.int/news-room/fact-sheets/detail/assistive-technology

[bibr88-14713012221127359] Young R. Camic P. M. Tischler V. (2016). The impact of community-based arts and health interventions on cognition in people with dementia: A systematic literature review. Aging & Mental Health, 20(4), 337–351. 10.1080/13607863.2015.1011080 25683767

[bibr89-14713012221127359] Zhang Y. Cai J. An L. Hui F. Ren T. Ma H. Zhao Q. (2017). Does music therapy enhance behavioural and cognitive function in elderly dementia patients? A systematic review and meta-analysis. Ageing Research Reviews, 35, 1–11. 10.1016/j.arr.2016.12.003 28025173

